# LD-annot: A Bioinformatics Tool to Automatically Provide Candidate SNPs With Annotations for Genetically Linked Genes

**DOI:** 10.3389/fgene.2019.01192

**Published:** 2019-11-26

**Authors:** Julien Prunier, Audrey Lemaçon, Alexandre Bastien, Mohsen Jafarikia, Ilga Porth, Claude Robert, Arnaud Droit

**Affiliations:** ^1^Genomics Center, Centre Hospitalier Universitaire de Québec–Université Laval Research Center, Quebec, QC, Canada; ^2^Forestry Research Centre, Forestry Department, Université Laval, Quebec, QC, Canada; ^3^Faculty of Agricultural and Food Science, Université Laval, Quebec, QC, Canada; ^4^Canadian Centre for Swine Improvement, Ottawa, ON, Canada; ^5^Department of Animal Biosciences, University of Guelph, Guelph, ON, Canada

**Keywords:** linkage disequilibrium, candidate SNP, SNP annotation, bioinformatics tool, variant call format (VCF), SNP chip analyses

## Abstract

A multitude of model and non-model species studies have now taken full advantage of powerful high-throughput genotyping advances such as SNP arrays and genotyping-by-sequencing (GBS) technology to investigate the genetic basis of trait variation. However, due to incomplete genome coverage by these technologies, the identified SNPs are likely in linkage disequilibrium (LD) with the causal polymorphisms, rather than be causal themselves. In addition, researchers could benefit from annotations for the identified candidate SNPs and, simultaneously, for all neighboring genes in genetic linkage. In such case, LD extent estimation surrounding the candidate SNPs is required to determine the regions encompassing genes of interest. We describe here an automated pipeline, “LD-annot,” designed to delineate specific regions of interest for a given experiment and candidate polymorphisms on the basis of LD extent, and furthermore, provide annotations for all genes within such regions. LD-annot uses standard file formats, bioinformatics tools, and languages to provide identifiers, coordinates, and annotations for genes in genetic linkage with each candidate polymorphism. Although the focus lies upon SNP arrays and GBS data as they are being routinely deployed, this pipeline can be applied to a variety of datasets as long as genotypic data are available for a high number of polymorphisms and formatted into a vcf file. A checkpoint procedure in the pipeline allows to test several threshold values for linkage without having to rerun the entire pipeline, thus saving the user computational time and resources. We applied this new pipeline to four different sample sets: two breeding populations GBS datasets, one within-pedigree SNP set coming from whole genome sequencing (WGS), and a very large multi-varieties SNP dataset obtained from WGS, representing variable sample sizes, and numbers of polymorphisms. LD-annot performed within minutes, even when very high numbers of polymorphisms are investigated and thus will efficiently assist research efforts aimed at identifying biologically meaningful genetic polymorphisms underlying phenotypic variation. LD-annot tool is available under a GPL license from https://github.com/ArnaudDroitLab/LD-annot.

## Introduction

The progress in molecular technologies enabled the study of genetic variants at the genome level, in both model and non-model species, such as Genome-Wide Association Studies (GWAS) identifying genetic variants likely involved in variation of interesting quantitative traits or in adaptation to environmental stress. Among those molecular techniques, SNP genotyping chips and genotyping-by-sequencing (GBS) approaches [also addressing the related reduction site-associated DNA sequencing (RADseq) in this paper] are often deployed to efficiently screen genomes at the population level and test for relationships between genetic polymorphisms and either quantitative characteristics or environmental conditions (i.e. Keller et al., 2013; Narum et al., 2013; Sonah et al., 2015; Carter et al., 2018; Torkamaneh et al., 2018;). GBS is based on sequencing genome subparts using restriction enzymes and insert size selection (Elshire et al., 2011) and yields thousands of genetic variants randomly distributed over the genome. SNP genotyping chips are based on allele-specific hybridization and traditionally include SNPs previously identified and selected to be regularly distributed across the genome (Carvalho et al., 2007; Bai et al., 2018). Both techniques usually result in thousands of SNPs successfully genotyped.

Research projects based on either of these variant detection approaches often investigate the genomic basis of trait variations related to agronomic performance in cultivated plants or animals (Carter et al., 2018; Torkamaneh et al., 2018;), the dispersion of invasive species (White et al., 2013; Roe et al., 2018), or species’ adaptation (Hess et al., 2012; Keller et al., 2013), for instance. Such studies typically use regression models to select candidate SNPs presenting significant trait variations between distinct genotypic classes. However, these polymorphisms might not be directly responsible for phenotypic variations but in linkage disequilibrium (LD) with larger genomic regions encompassing untested genetic variants that might be truly causal for the studied phenotypic variation.

LD is the non-random assortment of alleles between neighboring loci due to the short physical distance limiting recombination between them during meiosis. This phenomenon results in a systemic association between alleles of the same parental origin. For biallelic loci, LD is often estimated using the correlation coefficient (denoted *r* ^2^) between two alleles at two different loci (Hill and Robertson 1968). This estimate varies with the recombination coefficient which is a function of physical distance between markers (Hill and Robertson 1968). However, the recombination coefficient actually fluctuates along the genome, with regions known to present lower recombination coefficients than others, such as centromeric regions for instance (Smith et al., 2005). In addition, *r* ^2^ is also impacted by inbreeding which results in lower genetic diversity that in turn leads to homozygosity hiding recombination events. Hence, *r* ^2^ also varies between populations according to population demographic history (Reich et al., 2001), even within species. Similarly, the *r* ^2^ estimator presents a variability related to allele frequencies (minor allele frequency, MAF) (VanLiere and Rosenberg 2008) or sample size effect (Jorgenson and Witte 2006). Despite its limitations, the *r* ^2^ estimate remains largely used and most interesting when scanning GWAS results, for instance, since the correlation between two SNPs is still indicative of a mathematical link (Bush and Moore 2012), either reflecting a true low recombination rate between them or not.

Candidate polymorphisms, identified from GWAS or *F*_ST_-based outlier analyses for instance, most often need to be further studied with additional approaches such as gene expression profiling among individuals with contrasting trait expression or genetic engineering for instance, to corroborate these variants’ involvement in trait variation (Ermann and Glimcher 2012). In these regards, annotations of genes encompassing or overlapping DNA segments harboring SNPs in LD with these candidate ones (referred as genes in genetic linkage with candidate SNPs in this paper) are crucial to support their biological significance and help prioritize subsequent investigations. Given the *r* ^2^ variability among populations and markers subsets, estimating an experiment-specific LD on both sides of one candidate SNP is an adequate procedure to find the nearby genes that are genetically linked to this candidate and select significant annotations. Even though a number of softwares and packages dedicated to genomic polymorphisms annotation already exist (Wang et al., 2010; Rope et al., 2011; Cingolani et al., 2012), they either only consider the sequences encompassing the candidate SNPs (Wang et al., 2010; Cingolani et al., 2012) or use LD estimates from a different population, usually a population of reference such HapMapII or the 1000 Genomes Project in Humans (Johnson et al., 2008; Machiella and Chanock 2015), thus leading to limited or biased results. Furthermore, candidate polymorphisms found lying outside gene sequence boundaries are often annotated using the closest gene annotation in non-human organisms, without estimating in the specific experiment the genomic regions in genetic linkage with those (e.g. Stanton-Geddes et al., 2013). Thus, we developed a new bioinformatics annotation tool that estimates LD in order to gather annotations from regions genetically linked to candidate polymorphisms, thus strengthening their potential and help prioritizing them for further analyses.

## Materials and Methods

### Tested Datasets

When studying relationships between genetic markers and quantitative traits, research efforts usually involve testing and genotyping (1) hundreds to thousands of outbred individuals from natural populations, or (2) the progeny of a controlled cross between two individuals differing widely (i.e. segregating) for the trait of interest. In the first approach, individuals are sampled and later phenotyped in controlled and uniform conditions to perform a GWAS identifying candidate polymorphisms. In the second approach, a progeny is also assessed in controlled and uniform conditions, and the co-segregation of alleles and trait values allows to identify candidate SNPs. Both approaches have different assumptions regarding the levels of LD; average LD is usually moderate to low in association tests while very high in F1 progenies where many candidate SNPs are found in complete or nearly complete LD. Here, we tested our annotation pipeline with four different datasets to investigate a wide range of expected LD levels, originating from: (1) a domesticated animal, (2) a domesticated plant, and (3) a wild insect. These sets also varied in sampling size, numbers of tested SNPs, and candidate SNPs, thus further allowing to evaluate the pipeline’s performance.

#### Domesticated Species Datasets

**Figure 1:**
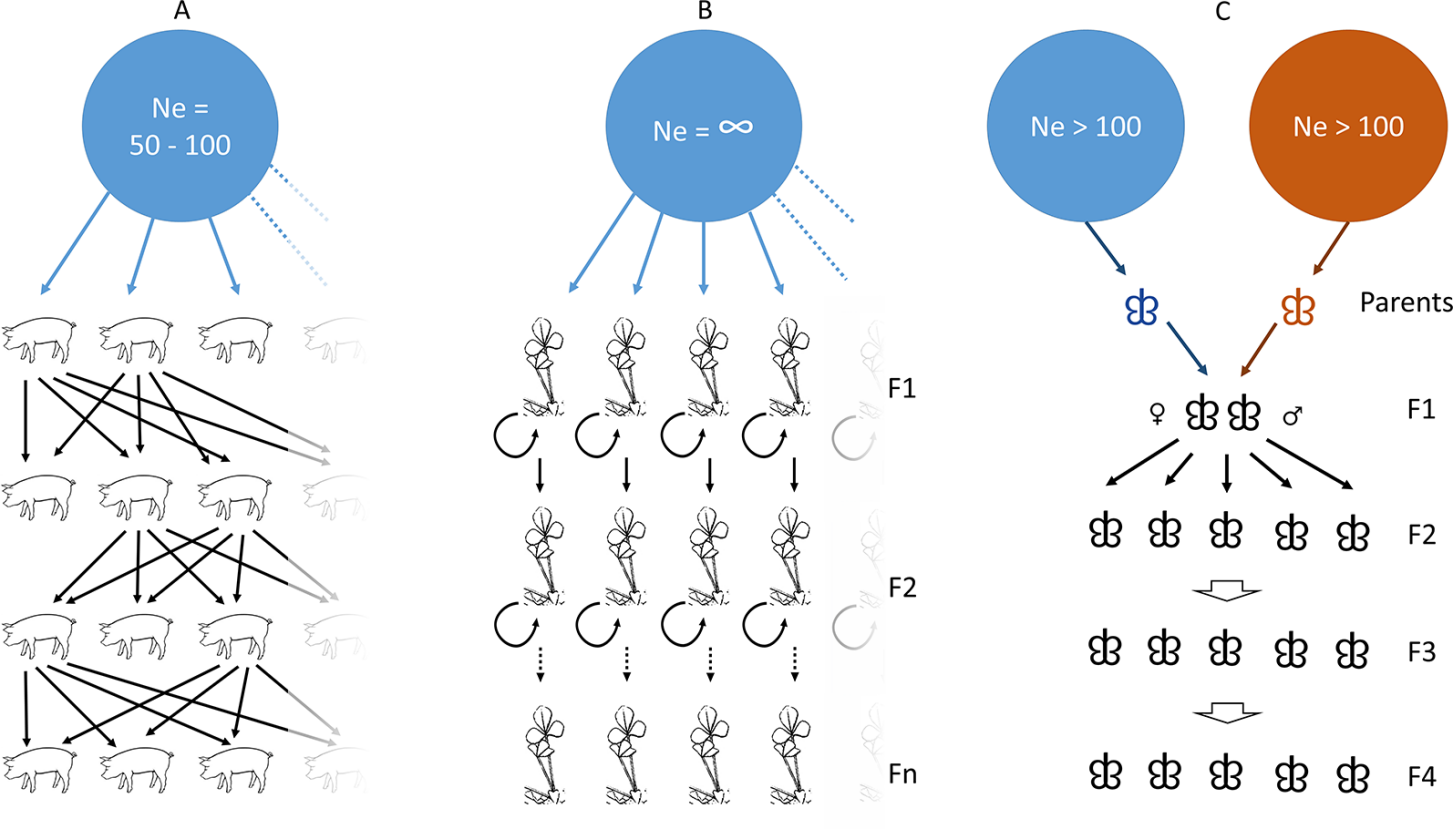
Population and kinship history for the three types of datasets used as study cases. **(A)** the pig case in which trait-based genetic selection has been performed for centuries from a large ancestral population many generations ago; **(B)** the *Medicago* case in which inbred lines have been obtained from self-crossing of individuals originating from a very large population; **(C)** the Asian gypsy moth case where an introgressed progeny was obtained from mating between a flying individual and a non-flying individual, repeated over few generations.

We applied our tool to annotate GWAS results in *Sus scrofa domesticus* which is characterized by high LD levels due to hundreds of years of selection to improve performance. This GWAS tested GBS data for association with meat quality (Prunier, Droit, Robert et al. unpublished) and was based on the genotyping of 196 individuals coming from two different breeding companies selecting sires and dams after each generation to improve meat quality in the Duroc pig breed (**Figure 1A**). The association tests yielded 199 candidate SNPs spread over the 18 autosomal chromosomes.

Even though the main focus of the present study is on GBS and SNP-array datasets, we also tested a dataset of 14,374,088 SNPs obtained from whole genome sequencing of the plant model *Medicago truncatula* varieties. These were investigated using GWAS for candidate genes involved in agronomic trait variations based on 226 accessions and representing as many inbred lines (Stanton-Geddes et al., 2013) (**Figure 1B**). The association study led to the identification of 1,537 candidate SNPs likely involved in variation of plant height or flowering timing, among other traits, and distributed over *Medicago*’s eight chromosomes. In order to run our pipeline, this publicly available dataset (www.medicagohapmap.org) was converted into a vcf file using bash commands and we tested both the entire set of SNPs and a set of SNPs with a minor allele frequency higher than 5%, yielding a total of 593,614 SNPs.

#### Wild Species Dataset

While three previous datasets were related to organisms with well described genomes, we finally assessed LD-annot capability to annotate candidate SNPs in a non-model, namely *Lymantria dispar* spp. This moth is an invasive species in North American forests as their caterpillars can successfully feed on foliage of numerous tree species (polyphagy) and therefore can damage vast tree plantations and natural forests. The co-segregation of SNP alleles and flying capabilities was followed over four generations (F2–F5) in this line resulting from the mating between a fully flying individual and a flightless individual in this species complex (**Figure 1C**). This analysis yielded a total of 250 SNPs possibly related to the moth’s ability to fly.

### Implementation

**Figure 2:**
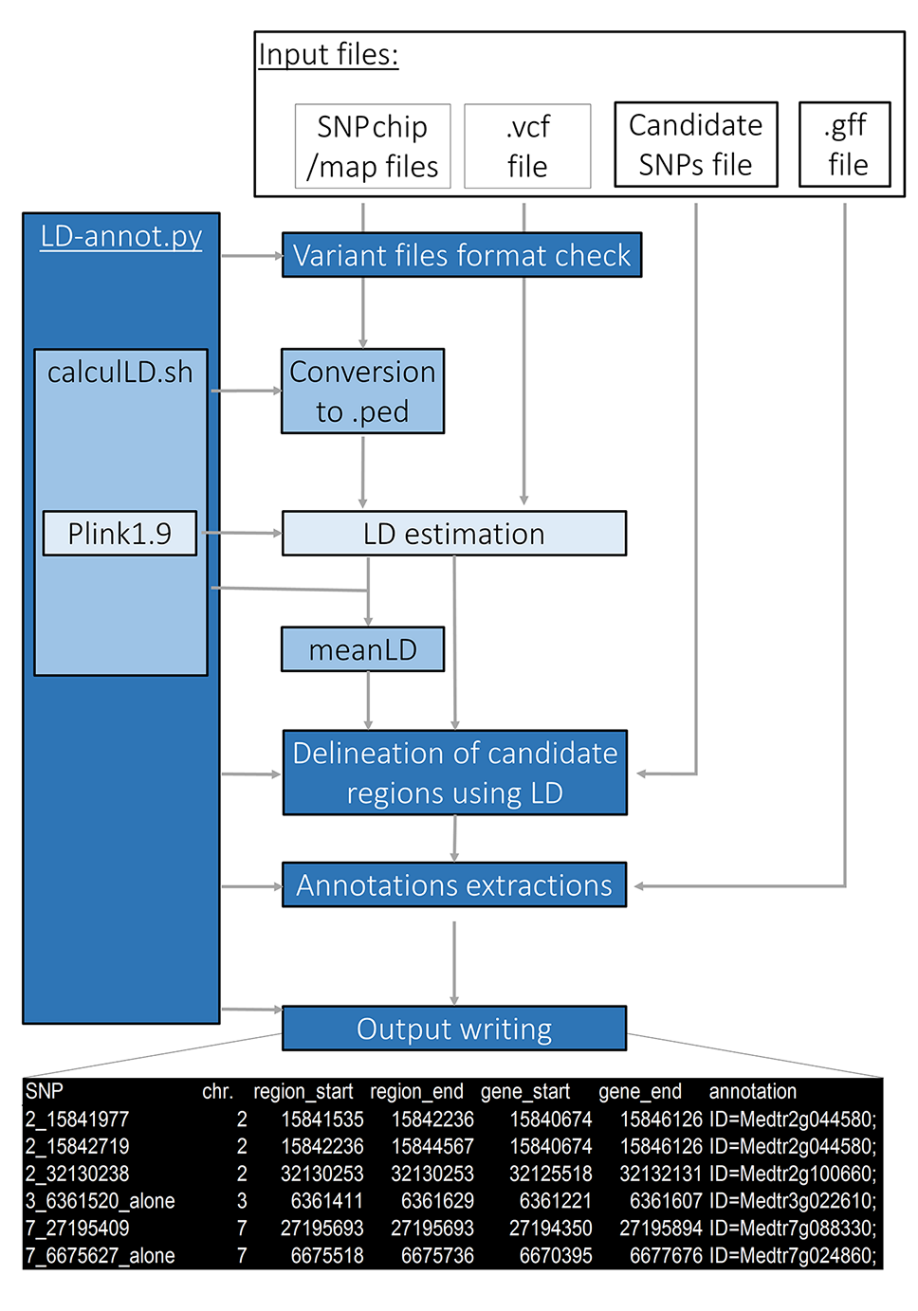
LD-annot overview. The LD-annot.py script is the master script that checks file format and calls a bash script for format conversion and PLINK LD estimation, and afterward calculates average LD and linked regions boundaries and gathers annotations for linked genes. At the bottom, an example header of the output file is presented.

The LD-annot pipeline efficiently integrates a public package as well as new bash and python scripts to import SNP-array data, estimate SNP-specific genomic regions genetically linked to candidate SNP and extract corresponding gene annotations (**Figure 2**). It can be deployed on any Unix-based (or bash developer mode on Windows OS) following installation steps described here: https://github.com/ArnaudDroitLab/LD-annot/blob/master/README.md.

LD-annot uses the public package PLINK1.9 to calculate LD (*r* ^2^) levels. The user must define an *r* ^2^ threshold for limiting the region surrounding a candidate SNP in which annotations will be extracted, i.e. only polymorphisms linked to one candidate polymorphism with a LD value superior to this threshold will be considered to delineate the region of interest (**Figure 2**). The pipeline includes a format check of input files and a checkpoint procedure. The latter allows to restart the analysis with different thresholds for *r* ^2^ for instance, without rerunning the format checks nor pairwise LD calculations, thus avoiding to run all steps and reducing the time for the analysis.

### Command and Parameters

The pipeline is launched using only a single command line containing the parameters and paths for input files. In addition, LD-annot.py calls a bash script (calculLD.sh) that must be placed in the same folder. The command using vcf format input file is:


python3 LD-annot.py geno.vcf annot.gff3 candidate\ type thr output

while the command using SNP-array input file is:


python3 LD-annot.py PathToSnpFiles annot.gff3\ candidate type thr output SNP_Map

where “type” is the feature (mRNA, CDS, gene), “thr” is the threshold for *r* ^2^, and “SNP_Map” is a txt file providing chromosome and position identifiers for each SNP included on the SNP-array.

### Inputs

The LD-annot pipeline is based on three different inputs.

The first input contains all genotypes for the studied population; this file is usually in vcf format obtained from a variant caller [Haplotypecaller or Platypus, for instance (DePristo et al., 2011; Rimmer et al., 2014)] for next-generation genotyping such as GBS data, or a folder including all individuals’ genotypes in the case of SNP-array genotyping. In the latter case, genotyping is usually spread over txt files, one for each individual, which contain polymorphisms names and genotypes after 12 lines of comments and headers. In the case of GBS data, the vcf file is directly converted by PLINK1.9 before running LD calculations. In the case of SNP-array data, a formatting step is performed before LD calculations using PLINK1.9. This bash script gathers all individuals’ genotypes included in the designated folder and converts this information into a .ped, .map, and .fam files for PLINK1.9 by making use of an additional input file providing the chromosome and position for each SNP on the SNP-array. Afterward, .ped files are converted to .bed files to save memory space and running time for both types of data, and *r* ^2^ are then calculated using PLINK1.9 (**Figure 2**).

The annotation file is a text file respecting a gff-like format (gff, gtf, or gff3) including the chromosome number/name in the first column, the feature in the third column (CDS, mRNA, exon), the starting and ending positions in respectively the fourth and fifth columns, and the annotation (= attributes) in the last column.

Finally, the third file contains the list of candidate SNPs with chromosome name in the first column, position in the second column, and SNP_ID in the third column (not required).

Note that the chromosome identification should be consistent among the various files; the number may often be prefixed with a “chr” or not. As this is the most likely source of errors and incompatibility, the format checking step generates error messages pointing at corrupt files and probable causes.

### Linkage Calculation and Annotation Extraction

Linkage disequilibrium is estimated using the *r* ^2^ correlation score calculated using PLINK for genotyped SNPs located on the same chromosome in linkage for *r* ^2^ > 0.4. This low threshold is defined as the lowest one that a user may select. The threshold defined by the user is used later in the pipeline when estimating an average distance in linkage with candidate SNPs according to this threshold, and during delineation of genomic regions in linkage with each candidate SNP for annotations extraction.

Based on the LD calculations previously computed and the *r* ^2^ threshold set by the user, annotations from a .gff/.gff3/.gtf-type file are then gathered to create an annotation file for each candidate variant. A “.gff/.gff3/.gtf” file usually includes annotations for different features (mRNA, CDS, exon, gene) which represents a hierarchical classification of the same genomic regions and thus results in some repetition of the information. According to the approaches deployed to annotate the reference genome, the level of its completeness or the biological question asked in the research, one might favor one over the other features. Thus, LD-annot offers an option to select the feature of interest and avoid redundancy of the information at the various levels (i.e. gene, mRNA, and exon), which also make it flexible to any feature that may be indicated in the annotations file.

After input format checking and *r* ^2^ calculations, the python script gathers chromosome, position, and annotation for the designated feature. Afterward, it makes a dictionary of “candidate” regions (chr, start, and end) around candidate SNPs by using the position of the foremost upward and downward SNPs in linkage with each one of those candidates according to *r* ^2^ threshold chosen by the user. However, a candidate SNP might not be surrounded by other genotyped SNPs because of true absence of polymorphisms (possibly in a specific sampling set) or low quality genotyping. In such cases, the average distance calculated earlier in the pipeline is used to delineate the region of interest around such candidates and an “alone” flag is added to the candidate SNP name in the output file. It should be noted that this average is a broad estimate and those results should be interpreted with caution given the *r* ^2^ variability along the genome, and the possibility of the non-Gaussian distribution of distances between SNPs in LD.

Finally, all annotated regions with the selected feature in .gff/.gff3/.gtf file that overlap the “candidate” region are included into an output file that provides: chromosome, candidate SNP position, region start and end positions, annotation start and end positions, and the annotation *per se*. According to the number of annotations overlapping the candidate region, a candidate SNP can be found several times in the output file.

## Results and Discussion

### LD-annot Performances

We assessed the performance of our tool through the analysis of the four datasets previously described and covering a large distribution in numbers of genotyped and candidate SNPs, and a variety of *r* ^2^ thresholds. The goal being to make this procedure amenable to researchers without coding skills nor access to high-performance infrastructures, we ran the pipeline using a common laptop computer with 4CPU cores and 8 Gbytes of RAM.

**Figure 3:**
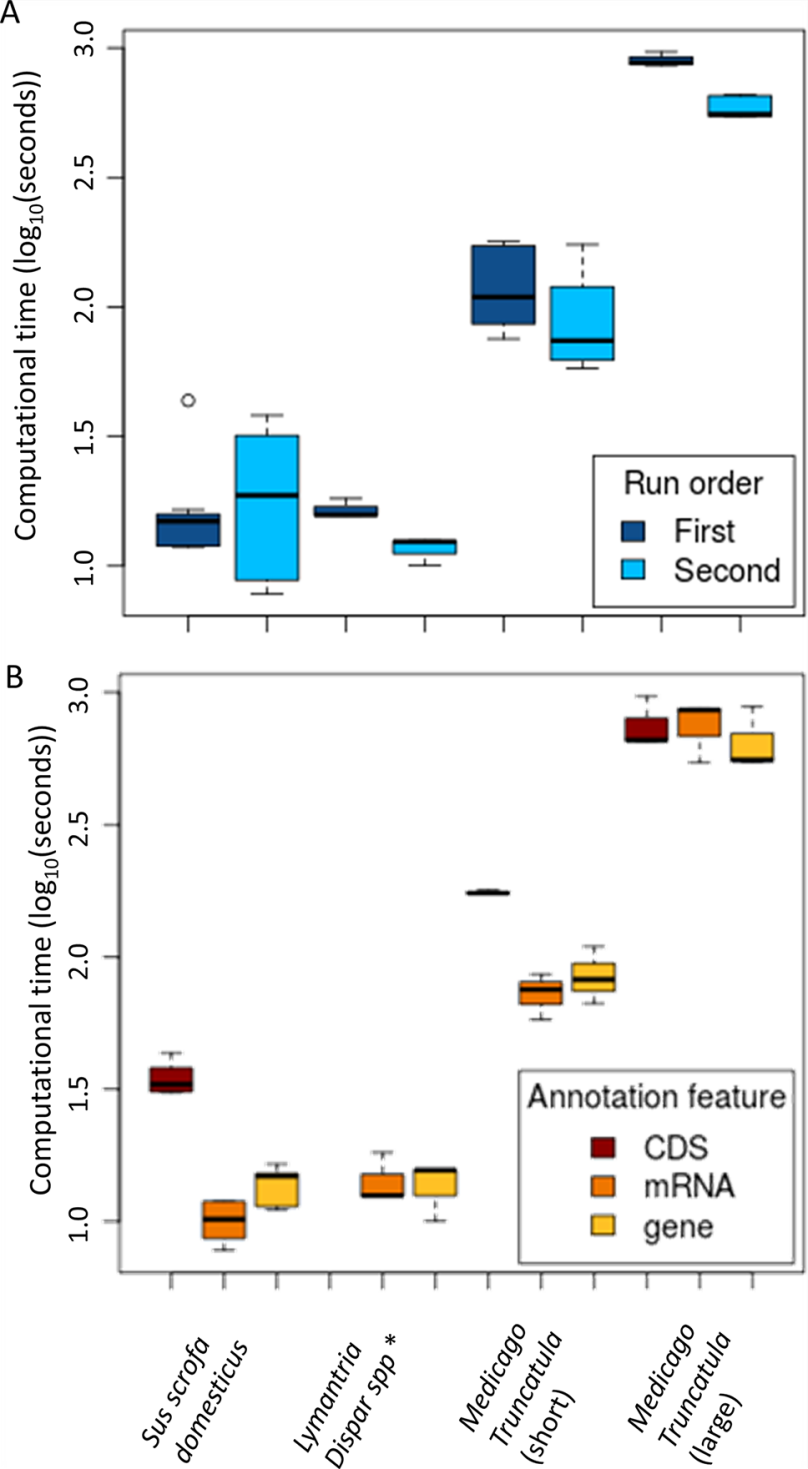
Pipeline performances according to the run number **(A)** and the type of annotated features **(B)**. **(A)** LD-annot involves a checkpoint procedure that does not require rerunning each step when testing several LD thresholds, which results in shorter turnover of analysis after its first run. **(B)** The type of feature has an impact on the time for analysis since mRNA and CDS features are usually more complex than gene features in an annotation file. *Note that no CDS annotations were available for the *Lymantria dispar* genome.

As expected, there was a significant correlation between the number of variants included in the analysis and the processing time (ANOVA, *p* < 2e-16; **Figure 3**). However, a single analysis never exceeded 16.1 min despite the very large SNP set (> 14M SNPs) originating from *Medicago* (**Table 1**). In such case, making use of the checkpoint feature allowed to reduce the computational time from 16.1 min to less than 10 (**Figure 3A**). As datasets are always increasing in size with technological progress and the usual need to test several *r* ^2^ thresholds, we believe the checkpoint procedure will be beneficial to the genomics research community.

**Table 1:** LD-annot time analysis according to the sizes of SNP sets and candidate SNP sets.

Dataset*	Total SNPs set size	Candidate SNP number	Time (s)	*r* ^2^ threshold	Average distance (bp)^†^
*Sus1*	54,712	199	18.3	0.7	50494
*Sus1*	54,712	199	19.3	0.9	18000
*Sus2*	54,712	199	20.0	0.7	53614
*Sus2*	54,712	199	21.0	0.9	17430
*Lymantria*	321,868	250	13.5	0.7	6191
*Lymantria*	321,868	250	14.0	0.9	4620
*Medicago*	593,614	1,536	109.7	0.7	706
*Medicago*	593,614	1,536	110.6	0.9	601
*Medic-large*	14,374,089	1,536	581.6	0.7	44
*Medic-large*	14,374,089	1,536	692.5	0.9	33

*Sus1 and Sus2: the two pig genotyping-by-sequencing datasets; Lymantria: the gypsy moth SNP set; Medicago: the public Medicago dataset after filtering for low minor allele frequencies; Medic-large: the entire SNP set for Medicago (Stanton-Geddes et al., 2013).

^†^Average distance between a pair of SNPs in linkage disequilibrium according to the threshold for r^2^ estimated from all SNPs in the dataset.

Another factor impacting the analysis time is the size of the annotation file and particularly the type of feature specified by the user in the command line. Annotation files (.gff/.gff3/.gtf) typically harbor more annotation lines in the “CDS” feature than for “gene” or “mRNA.” As a result, the analyses were significantly longer when searching for “CDS” feature annotations (ANOVA, *p* = 0.0137; **Figure 3B**). In line with this trend, regions linked to candidate SNPs extended when the *r* ^2^ threshold increased, resulting in an increasing number of annotations and time length for the analysis, although the difference was not significant.

### Average Distance

The LD-annot pipeline calculates an average distance (in bp) separating two SNPs in LD according to the specified *r* ^2^ threshold across the whole dataset. This distance is later used to delineate a linked region around a candidate SNP (the average distance on both sides) when there is no surrounding genotyped SNPs. This distance is a function of inbreeding as illustrated by our datasets where the higher the original effective population size, the shorter is the distance in LD. Even within the pig species, the pedigree denoted *Sus1* generally presented shorter distances than* Sus2* pedigree which was developed from a smaller effective population of sires and dams.

This distance is also varying according to the number of genotyped SNPs which is related to the occurrence of rare SNPs that tend to present lower *r* ^2^ values than more common SNPs (Pritchard and Przeworski 2001; Pe’er et al., 2006). As a result, removing SNPs with minor allele frequency <0.05 resulted in a sizable increase in distances (up to 18-fold) when testing the *Medicago* SNP set.

When genotyping a sample set using GBS approach, the SNP distribution over the genome is not controlled and the proportion of the genome interrogated by the genotyping is often an important question for researchers. The average distance provided by the tool can further be used to broadly estimate the genome coverage given the *r* ^2^ thresholds. For instance, using 54,712 SNPs in the *Sus1* pedigree allowed to investigate the entire 2.4Gb *Sus scrofa* genome with *r* ^2^ > 0.7, but 82% and only 40% of this genome with *r* ^2^ > 0.8 and 0.9, respectively. The same SNP set in the *Sus2* pedigree allowed to investigate 100, 87, and 38% of the genome with *r* ^2^ > 0.7, 0.8, and 0.9, respectively. However, these coverage values should be seen as broad estimates and, therefore, interpreted with caution given *r* ^2^ variability across the genome.

### Why Not Consider Only the Closest Gene?

**Figure 4:**
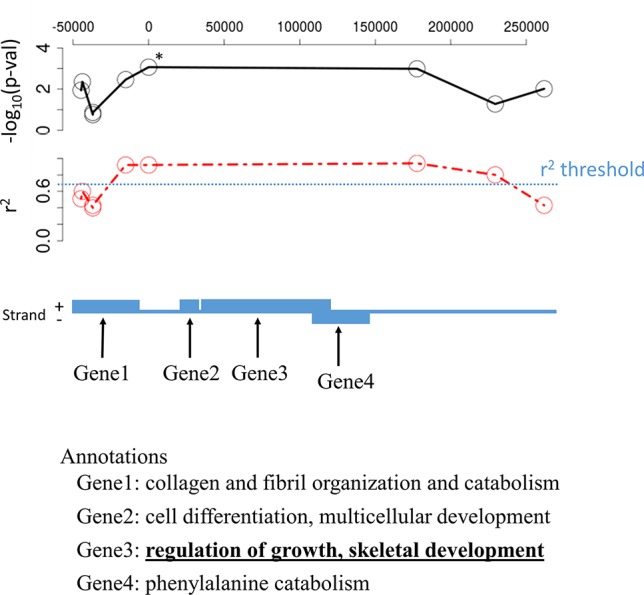
Illustration of one candidate SNP likely involved in pig meat quality that is genetically linked to four different genes; note that the most biologically meaningful is not the closest one but of the third order. The candidate SNP is at the position “0” upon the chromosome and marked with an asterisk; –log_10_(p-val) is the p-value for the association test between allelic variation and meat quality; *r* ^2^ is the correlation coefficient calculated in the dataset (red line) using PLINK and the specified threshold for linkage was 0.7 (blue line).

Selecting annotations associated with a candidate polymorphism is usually accomplished using the proximity criteria, in other words, the gene including the SNP in its sequence or the closest gene for non-coding SNP is often seen as the relevant one (e.g. Stanton-Geddes et al., 2013). However, other remote genes might be in genetic linkage with the candidate SNP while not presenting SNP in the studied SNP set, which does not allow to test their association *per se*. Even when presenting SNPs, these genes may have been missed because of too many missing genotypes or too low minor allele frequency for a specific locus which, in turn, did not permit to significantly detect them as candidate SNPs. For instance, when using LD-annot in *Sus scrofa*, we found a total of 334 genes in genetic linkage with only 176 of the candidate SNPs while the remaining candidate SNPs were not linked to any genes using an *r* ^2^ threshold >0.7. We even observed six cases of annotations for distant genes (second or third order of the closest genes and still in LD with the candidate SNP using *r* ^2^ > 0.9) that were in fact more informative with regards to the trait of interest than the closest one (**Figure 4**).

Contrastingly, the closest gene might be far away and not genetically linked with the candidate SNP which could lead to biased interpretation, particularly when performing enrichment analyses. In *Medicago*, over the 1,536 candidate SNPs that were annotated using the closest gene (Stanton-Geddes et al., 2013), only 541 SNPs were actually genetically linked with their target gene (*r* ^2^ > 0.7). On the other hand, 40 candidate SNPs were genetically linked with two genes, and 62 annotated genes were linked to more than one candidate SNP (**Supplementary Table 1**), hence showing the importance of taking into account the LD when looking at annotations supporting the importance of a candidate SNP.

In the case of progenies study (gypsy moth case), the LD level is very high which resulted in blocks of several candidate SNPs genetically linked together, thus defining large regions possibly encompassing several genes. However, only 100 SNPs were in linkage with 64 genes (*r* ^2^ > 0.9) among the 250 candidate SNPs spread over 103 contigs. Despite the high level of LD and that all scaffolds harboring a candidate SNP were also encompassing one gene at the very least (2.39 genes in average), some candidate SNPs were not found in genetically linked with any gene. The distribution of recombination rates was not continuous as expected given the low number of individuals and generations, and LD breakpoints were observed along scaffolds. Thus, a SNP might be relatively close to a gene but still not representing it. Altogether, these results illustrate the need to evaluate the experiment-specific LD surrounding candidate SNPs when employing genes to annotate and prioritize these for further investigations, and understand the mechanisms underlying their association with trait variation.

## Conclusion

The LD-annot tool yields supporting lines of evidence to help identify biologically meaningful genetic polymorphisms underlying phenotypic variation. It can be used with any sort of annotations and polymorphism data as long as the input format matches either SNP-chips or vcf files. One can obtain annotations for repeats or specific methylation sites, for instance, and use this tool to identify those features that are statistically linked to candidate SNPs for a given sampling.

## Data Availability Statement

Medicago data can be found in Stanton-Geddes et al. 2013. Data generated in this study are included in the article/**Supplementary Material**. Scripts are available at: https://github.com/ArnaudDroitLab/LD-annot/.

## Author Contributions

JP developed and coded the bioinformatics tool with help from AL and AB, and tested it using the various datasets. MJ gathered the pig meat quality measurements. IP obtained the funding allowing to sequence the gypsy moth pedigree and JP identified candidate SNPs for flight in this pedigree. CR and AD obtained the funding to sequence pig individuals and support the bioinformatics tool development. All co-authors read and edited the manuscript.

## Funding

This work, including genotyping-by-sequencing data in pig, has been funded by the “chips-for-better-chops” project financially supported by Genome Canada (Genomic Applications Partnership Program), the Canadian Centre for Swine Improvement, FastGenetics Inc. and Olymel Inc. Asian gypsy moth sequence data originated from the BioSafe project financially supported by Genome Canada, Genome BC and Genome Quebec and the Canadian Food Inspection Agency.

## Conflict of Interest

The authors declare that this study received funding from FastGenetics Inc. and Olymel Inc. which also provided meat quality data for the pigs’ use-cases. The authors declare that the research was conducted in the absence of any commercial or financial relationships that could be construed as a potential conflict of interest.
